# Bridging Body and Mind: A Collaborative Physical Health Curriculum for Psychiatrists

**DOI:** 10.1192/bjo.2026.11291

**Published:** 2026-06-30

**Authors:** Emily Pettifor, Sofia Frangiamore, Burhan Khan

**Affiliations:** 1 Kent and Medway Mental Health NHS Trust, Dartford, United Kingdom; 2 Dartford and Gravesham NHS Trust, Dartford, United Kingdom

## Abstract

**Aims:**

To address gaps in physical health teaching for psychiatric doctors, we hypothesised that a structured programme delivered collaboratively across trusts would improve confidence and knowledge in managing acute and chronic medical conditions. Psychiatric doctors frequently encounter physical health issues with their patients, yet ongoing training opportunities are limited. Feedback from trust resident-senior forums highlighted a need for teaching on medical emergencies and chronic disease management.

**Methods:**

A curriculum was developed covering 12 core topics, including medical emergencies in psychiatry, cardiovascular health and integrated care. Sessions are delivered bi-monthly within the trust’s continuing professional development programme for higher trainees and consultants, with additional invitations sent to core trainees and IMTs for these talks. Teaching is led by resident doctors and consultants in adult medicine, with feedback provided by attendees for their portfolios. Teaching materials can be offered for review by a psychiatry trainee beforehand to ensure relevance to mental health settings and discuss further. Staff attendance is encouraged through induction packs and circulated schedules.

**Results:**

Sessions have been well attended, with on average 85 staff (range 67–110), including both resident doctors and consultants. Feedback has been overwhelmingly positive, citing sessions as “practical”, “relevant”, and “useful” for out-of-hours support. Participants valued guidance on identifying alarming signs, understanding hospital expectations from referrals and insight into likely treatment for their patients. One staff member noted they would “reach for these slides during on-calls” as an additional available resource. It was also highlighted that there could be misconceptions between hospital and psychiatric teams regarding available tools and resources. This shows the importance of shared understanding to improve patient care.

**Conclusion:**

High engagement and positive feedback demonstrate the need for ongoing physical health teaching in psychiatry. This programme exemplifies how cross-trust collaboration can address learning gaps and could be replicated in other regions to enhance patient safety and clinician confidence.

